# Supplementary material for: Potential global distribution of Setaria italica, an important species for dryland agriculture in the context of climate change
Source: PLoS One. 2024 Apr 16;19(4):e0301751. doi: 10.1371/journal.pone.0301751 (PMC11020860; doi:10.1371/journal.pone.0301751)

PLOS ONE

Potential global distribution of Setaria italica , an important species for dryland agriculture in the context of climate change

--Manuscript Draft--

**Manuscript Number:**	PONE-D-23-42450
**Article Type:**	Research Article
**Full Title:**	Potential global distribution of Setaria italica , an important species for dryland agriculture in the context of climate change
**Short Title:**	Potential global distribution of Setaria italica for dryland agriculture in the context of climate change
**Corresponding Author:**	Yi Huang<br>Tibet University<br>Tibet, CHINA
**Keywords:**	Setaria italica; climate change; potential distribution; MaxEnt
**Abstract:**	Setaria italica (S. italica, Linnaeus, 1753) is a C-4 crop with characteristics of drought resistance, infertile tolerance, and wide adaptability. It is also a specialty crop for maintaining agricultural and economic stability in arid and barren regions of the world. However, the potential cultivation region suitable for S. italica under Current and Future Climatic Scenarios still needs to be discovered. Predicting the potential global geographic distribution of S. italica and clarifying its ecological requirement will help promote agricultural development. It also plays a crucial role in maintaining people's lives and health and further solving the global food crisis. In this study, with a combination of environmental factor variables, we predicted the potential global geographic distribution of S. italica based on 3154 global distribution records through the Maxent model and ArcGIS software; We assessed constraints on its potential distribution by using the contribution of environmental factor variables. We evaluated the prediction accuracy of the Maxent model using three approaches: AUC values, TSS values, and Kappa statistics. The results showed that the Maxent model had high prediction accuracy and the simulation results were also reliable; the total suitable habitats of S. italica is 5535×104 km2, which mainly included the United States (North America), Brazil (South America), Australia (Oceania), China, India (Asia), and the Russian Federation (Europe). The most suitable habitat of S. italica, was 522.64×104 km2, accounting for 9.44% of the total areas, mainly in the United States, India, the Russian Federation, China, and the like. Soil and precipitation (driest monthly precipitation, hottest seasonal precipitation) are the most important factors, limiting the potential distribution of S. italica. Compared with the modern potential distribution, we predict that the four future climate change scenarios will lead to varying degrees of reduction in the likely geographic ranges of S. italica. Our prediction shows that climate change may significantly affect the global future distribution pattern of S. italica cultivation, which could reshape the global S. italica production and trade patterns.
**Order of Authors:**	Jingtian Yang
	Xue Jiang
	Yunlong Ma
	Mei Liu
	Zixi Shama
	Jiayi Li
	Yi Huang
**Additional Information:**	
**Question**	**Response**
**Financial Disclosure**	Yes

Powered by Editorial Manager® and ProduXion Manager® from Aries Systems Corporation

Enter a financial disclosure statement that describes the sources of funding for the work included in this submission. Review the submission guidelines for detailed requirements. View published research articles from *PLOS ONE* for specific examples.<br><br>This statement is required for submission and **will appear in the published article** if the submission is accepted. Please make sure it is accurate.<br><br>**Funded studies**<br>Enter a statement with the following details:<br>• Initials of the authors who received each award<br>• Grant numbers awarded to each author<br>• The full name of each funder<br>• URL of each funder website<br>• Did the sponsors or funders play any role in the study design, data collection and analysis, decision to publish, or preparation of the manuscript?<br><br>Did you receive funding for this work?	
Please add funding details.<br>as follow-up to "**Financial Disclosure**<br><br>Enter a financial disclosure statement that describes the sources of funding for the work included in this submission. Review the submission guidelines for detailed requirements. View published research articles from *PLOS ONE* for specific examples.<br><br>This statement is required for submission and **will appear in the published article** if the submission is accepted. Please make sure it is accurate.	The Scientific research initiation project of Mianyang Normal University (QD2019A13, QD2021A37 and QD2023A01), the Funding of the Open Project from the Ecological Security and Protection Key Laboratory of Sichuan Province (ESP1608, ESP2008, ESP2201 and ESP2204), the Sichuan Provincial Education Department Scientific Research Project (15ZB0283), the Sichuan Provincial Science and Technology Department Project (2023NSFSC0750).

Powered by Editorial Manager® and ProduXion Manager® from Aries Systems Corporation

**Funded studies**<br>Enter a statement with the following details:<br>• Initials of the authors who received each award<br>• Grant numbers awarded to each author<br>• The full name of each funder<br>• URL of each funder website<br>• Did the sponsors or funders play any role in the study design, data collection and analysis, decision to publish, or preparation of the manuscript?<br><br>Did you receive funding for this work?"	
Please select the country of your main research funder (please select carefully as in some cases this is used in fee calculation).<br>as follow-up to "**Financial Disclosure**<br><br>Enter a financial disclosure statement that describes the sources of funding for the work included in this submission. Review the submission guidelines for detailed requirements. View published research articles from *PLOS ONE* for specific examples.<br><br>This statement is required for submission and **will appear in the published article** if the submission is accepted. Please make sure it is accurate.<br><br>**Funded studies**<br>Enter a statement with the following details:<br>• Initials of the authors who received each award<br>• Grant numbers awarded to each author<br>• The full name of each funder<br>• URL of each funder website<br>• Did the sponsors or funders play any role in the study design, data collection and analysis, decision to publish, or preparation of the manuscript?<br><br>Did you receive funding for this work?"	CHINA - CN
**Competing Interests**	The authors have declared that no competing interests exist

Use the instructions below to enter a competing interest statement for this submission. On behalf of all authors, disclose any competing interests that could be perceived to bias this work—acknowledging all financial support and any other relevant financial or non-financial competing interests.<br><br>This statement is required for submission and **will appear in the published article** if the submission is accepted. Please make sure it is accurate and that any funding sources listed in your Funding Information later in the submission form are also declared in your Financial Disclosure statement.<br><br>View published research articles from *PLOS ONE* for specific examples.<br><br>**NO authors have competing interests**<br><br>Enter: *The authors have declared that no competing interests exist.*<br><br>**Authors with competing interests**<br><br>Enter competing interest details beginning with this statement:<br><br>*I have read the journal's policy and the authors of this manuscript have the following competing interests: [insert competing interests here]*<br><br>* typeset	
**Ethics Statement**<br><br>Enter an ethics statement for this submission. This statement is required if the study involved:<br><br>• Human participants<br>• Human specimens or tissue<br>• Vertebrate animals or cephalopods<br>• Vertebrate embryos or tissues	N

Powered by Editorial Manager® and ProduXion Manager® from Aries Systems Corporation

• Field research<br><br>Write "N/A" if the submission does not require an ethics statement.<br><br>General guidance is provided below. Consult the submission guidelines for detailed instructions. **Make sure that all information entered here is included in the Methods section of the manuscript.**	

**Format for specific study types**<br><br>**Human Subject Research (involving human participants and/or tissue)**<br>• Give the name of the institutional review board or ethics committee that approved the study<br>• Include the approval number and/or a statement indicating approval of this research<br>• Indicate the form of consent obtained (written/oral) or the reason that consent was not obtained (e.g. the data were analyzed anonymously)<br><br>**Animal Research (involving vertebrate animals, embryos or tissues)**<br>• Provide the name of the Institutional Animal Care and Use Committee (IACUC) or other relevant ethics board that reviewed the study protocol, and indicate whether they approved this research or granted a formal waiver of ethical approval<br>• Include an approval number if one was obtained<br>• If the study involved *non-human primates*, add *additional details* about animal welfare and steps taken to ameliorate suffering<br>• If anesthesia, euthanasia, or any kind of animal sacrifice is part of the study, include briefly which substances and/or methods were applied<br><br>**Field Research**<br><br>Include the following details if this study involves the collection of plant, animal, or other materials from a natural setting:<br>• Field permit number<br>• Name of the institution or relevant body that granted permission	
**Data Availability**<br><br>Authors are required to make all data underlying the findings described fully available, without restriction, and from the time of publication. PLOS allows rare exceptions to address legal and ethical concerns. See the PLOS Data Policy and FAQ for detailed information.	Yes - all data are fully available without restriction

Powered by Editorial Manager® and ProduXion Manager® from Aries Systems Corporation

A Data Availability Statement describing where the data can be found is required at submission. Your answers to this question constitute the Data Availability Statement and **will be published in the article**, if accepted.<br><br>**Important:** Stating 'data available on request from the author' is not sufficient. If your data are only available upon request, select 'No' for the first question and explain your exceptional situation in the text box.<br><br>Do the authors confirm that all data underlying the findings described in their manuscript are fully available without restriction?	
**Describe where the data may be found in full sentences. If you are copying our sample text, replace any instances of XXX with the appropriate details.**<br><br>• If the data are **held or will be held in a public repository**, include URLs, accession numbers or DOIs. If this information will only be available after acceptance, indicate this by ticking the box below. For example: *All XXX files are available from the XXX database (accession number(s) XXX, XXX.)*.<br>• If the data are all contained **within the manuscript and/or Supporting Information files**, enter the following: *All relevant data are within the manuscript and its Supporting Information files.*<br>• If neither of these applies but you are able to provide **details of access elsewhere**, with or without limitations, please do so. For example:<br><br>*Data cannot be shared publicly because of [XXX]. Data are available from the XXX Institutional Data Access / Ethics Committee (contact via XXX) for researchers who meet the criteria for access to confidential data.*<br><br>*The data underlying the results presented in the study are available from (include the name of the third party*	All relevant data are within the manuscript and its Supporting Information files.

and contact information or URL).<br>• This text is appropriate if the data are owned by a third party and authors do not have permission to share the data.<br><br>* typeset	
Additional data availability information:	

Manuscript Click here to access/download;Manuscript;Manuscript.docx

Potential global distribution of *Setaria italica*, an important species for dryland agriculture in the context of climate change

Jingtian Yang[1], Xue Jiang[2], Yunlong Ma[2], Mei Liu[1], Zixi Shama[1], Jiayi Li[1], Yi Huang[2,3*]

1 Ecological Security and Protection Key Laboratory of Sichuan Province, Mianyang Normal University, Mianyang 621000, China

2 Engineering Research Center for Forest and Grassland Disaster Prevention and Reduction, Mianyang normal university, Mianyang 621000, China

3 China College of Science, Tibet University, Lhasa 850012, China

*Correspondence: hyhy1232021@163.com (Y.H.)

Abstract: *Setaria italica* (*S. italica*, Linnaeus, 1753) is a C-4 crop with characteristics of drought resistance, infertile tolerance, and wide adaptability. It is also a specialty crop for maintaining agricultural and economic stability in arid and barren regions of the world. However, the potential cultivation region suitable for *S. italica* under Current and Future Climatic Scenarios still needs to be discovered. Predicting the potential global geographic distribution of *S. italica* and clarifying its ecological requirement will help promote agricultural development. It also plays a crucial role in maintaining people's lives and health and further solving the global food crisis. In this study, with a combination of environmental factor variables, we predicted the potential global geographic distribution of *S. italica* based on 3154 global distribution records through the Maxent model and ArcGIS software; We assessed constraints on its potential distribution by using the contribution of environmental factor variables. We evaluated the prediction accuracy of the Maxent model using three approaches: AUC values, TSS values, and Kappa statistics. The results showed that the Maxent model had high prediction accuracy and the simulation results were also reliable; the total suitable habitats of *S. italica* is 5535×10^4 km^2, which mainly included the United States (North America), Brazil (South America), Australia (Oceania), China, India (Asia), and the Russian Federation (Europe). The most suitable habitat of *S. italica*, was 522.64×10^4 km^2, accounting for 9.44% of the total areas, mainly in the United States, India, the Russian Federation, China, and the like. Soil and precipitation (driest monthly precipitation, hottest seasonal precipitation) are the most important factors, limiting the potential distribution of *S. italica*. Compared with the modern potential distribution, we predict that the four future climate change scenarios will lead to varying degrees of reduction in the likely geographic ranges of *S. italica*. Our prediction shows that climate change may significantly affect the global future distribution pattern of *S. italica* cultivation, which could reshape the global *S. italica* production and trade patterns.

Keywords: *Setaria italica*; climate change; potential distribution; MaxEnt

1. Introduction

The factors influencing crop growth, development, and distribution have been widely discussed and studied for decades and have become a hot scientific issue [8, 7, 20, 37]. Crops, as a distinctive plant resource, bear a profound connection to both social and agricultural advancements [8, 35].

Various determinants, encompassing climate, topography, hydrology, and soil composition, exert their influence on crop growth and development [7, 21]. Recent inquiries underscore that, amid these multifaceted influences, non-climatic factors predominantly govern short-term biological changes, with climate change emerging as the pivotal factor significantly impacting growth, development, and optimal distribution [6, 21, 33]. Widely acknowledged as a critical driver of agricultural progress, climate change finds consensus within the scientific community [7, 9, 37]. Research indicates that global warming substantially affects crop yields, posing a significant threat to global food security and sustainable development [2]. Conducting a trend analysis reveals the imperative to steadfastly pursue basic food self-sufficiency as a strategic measure to uphold international peace and security [36]. Consequently, a systematic exploration into the geographical distribution patterns of crops and their responses to climate change in the context of global warming assumes paramount importance. This investigation not only furnishes scientific evidence crucial for production layout and planting management but also establishes a foundational framework for regional sustainable development, ecological stewardship, and environmental governance. The implications of this exploration extend significantly towards ensuring global food security in the face of evolving climatic conditions.

Species Distribution Models (SDMs) have gained widespread use in examining the impact of climate change on the potential geographic range of species. These models play an increasingly pivotal role in forecasting species distribution by considering alterations in diverse environmental factors [11, 21]. Over ten distinct models, including Bioclim, Domain, GARP, MaxEnt, and others, have found applications across various domains such as plant conservation, utilization, and pest invasion [11, 12, 28-31, 41]. Notably, the MaxEnt model possesses several advantages compared to its counterparts: (1) singular distribution records suffice during the modeling process, (2) it accommodates both continuous and discrete variables, (3) its operational simplicity eliminates the need for intricate format conversions of species distribution and environmental data, (4) a minimal sample of species "presence" data yields highly effective simulations, and (5) its theoretical underpinnings are closely aligned with ecological principles, enhancing understanding of species suitability [7, 20, 18, 32]. Given the divergence in theoretical foundations among various models, their simulation and prediction performances exhibit considerable variation. MaxEnt, among these models, consistently demonstrates superior simulation outcomes [10, 33]. Consequently, the MaxEnt model emerges as particularly well-suited for evaluating the potential global distribution of *S. italica*, considering its robust simulation results and alignment with the ecological context, as elucidated above.

The species *S. italica*, an annual herb belonging to the Gramineae family, possesses characteristics akin to millet, as depicted in Figure 1. It stands as one of the earliest globally domesticated crops, demonstrating resilience to drought, infertility, and high temperatures [16, 44, 25, 42, 43]. Renowned for its nutritional value and enriched with natural active substances conferring hypoglycemic, hypolipidemic, and antioxidant properties, *S. italica* additionally serves as a noteworthy C4 biofuel source. This versatility is reflected in its processing into a myriad of food and industrial products, as illustrated in Figure 1d [4, 44]. As a paramount miscellaneous crop on the global stage, *S. italica* enjoys popularity due to its high yield and cost-effectiveness [1, 44]. Given the contemporary prevalence of high-protein dietary habits across a significant portion of the global population, the

integration of *S. italica* into diets becomes instrumental in fostering diverse and balanced nutritional intake [44]. In essence, the significance of *S. italica* extends beyond its economic contributions to encompass vital implications for maintaining human well-being and global food security. Despite its agricultural prominence, there exists a noteworthy gap in potential distribution studies for *S. italica*. Ecological modeling studies with consideration of environmental variables, as demonstrated in several existing works [7, 24, 35, 37], offer practical insights into predicting the potential suitable distribution of crops. However, the dearth of analogous studies for *S. italica* underscores the importance of research focused on delineating its current and future global suitable distribution. Such investigations hold promise not only for advancing agricultural development but also for addressing critical aspects of human life and health, thereby contributing meaningfully to resolving the ongoing global food crisis.

Fig. 1. Stages of *S. italica* Processing: (a) Unprocessed, (b) Harvesting, (c) After shelling, (d) Processed products.

Given the absence of dedicated studies on the potential geographical distribution of *S. italica*, employing the MaxEnt model emerges as a suitable approach. While numerous studies have applied various models to predict crop distributions, the specific considerations for *S. italica* have been notably absent. The cultivation of *S. italica* currently confronts practical challenges, including the unknown potential planting areas and the imperative to comprehend its distribution dynamics in response to climate change within the context of global warming.

This study aims to address these challenges by predicting the potential distribution of *S. italica* across different periods, offering a theoretical reference for cultivation practices. The research endeavors to unveil the present and future distribution patterns of *S. italica*, delineate trends in its potential distribution under the influence of global warming and put forth development strategies to address emerging challenges. Furthermore, the study explores the intricate relationship between the distribution of *S. italica* and climate change, as illustrated in Figure 2. By delving into these aspects, the research aims to contribute valuable insights for the theoretical underpinning of *S. italica* cultivation, facilitating informed decision-making in the face of uncertainties associated with global warming.

Fig. 2. Flowchart displaying the steps of the present study

2. Materials and methods

2.1 Species data sources

To obtain the occurrences of *S. italica*, we conducted field surveys and made queries in databases such as the Global Biodiversity Database (http://www.gbif.org/, last accessed on April 2023), China Plantwise (http://www.iplant.cn, last accessed on April 2023) and China Digital Herbarium (https://www.cvh.ac.cn, last accessed on April 2023)) [29, 41]. As a result, we compiled 3,154 distribution points of *S. italica* (Fig. 3).

Fig. 3. Global Occurrence records of *S. italica*

2.2 Sources of environmental variables

For our study, we gathered essential data from reputable sources to conduct a comprehensive analysis of the potential distribution of *S. italica*. Climate and digital elevation data for both current and

future emission scenarios were retrieved from the WorldClim database (DEM, http://www.worldclim.org//). Soil data, integral to our investigation, corresponds to the Coordinated World Soil Database (HWSD V1.2, https://www.fao.org/), ensuring a robust understanding of the soil characteristics. UV-B radiation data, a crucial environmental factor, was sourced from the global UV-B radiation dataset (gIUV, https://www.ufz.de/gluv/index.php).

To enhance the spatial context of our study, we incorporated world administrative maps obtained from the National Science and Technology Infrastructure System Science Data Center China via National Earth (http://www.geodata.cn). The integration of these datasets was achieved through the utilization of ArcGIS software (version 10.2), facilitating a unified representation at a 5 arc-minute resolution.

Acknowledging the potential interdependence of environmental variables, we conducted a Spearman correlation analysis as depicted in Figure 4. This analysis allowed us to discern correlations among the variables before incorporating them into the MaxEnt model. In instances where the correlation coefficient between two environmental factors exceeded or equaled 0.8, a judicious approach was taken to retain the variable contributing more significantly. As a result of this selection process, eight key environmental variables were identified and utilized in subsequent MaxEnt model runs, ensuring the model's robustness and effectiveness in predicting the potential distribution of *S. italica*.

Fig. 4. Heat map for correlation analysis of environmental variables

2.3 Model construction and evaluation

The MaxEnt modeling process involved importing distribution point and environmental factor data for *S. italica*, which were divided into a test set (25%) and a training set (75%) to ensure the accuracy of our predictions. To address the inherent challenge of false positives and negatives in species distribution models, rigorous evaluation metrics were employed to assess the usability and accuracy of the model [20, 33]. Commonly utilized theoretical evaluation metrics, including overall accuracy, sensitivity, specificity, AUC values, TSS values, and Kappa statistics, were considered. For enhanced credibility, this study selected AUC values, TSS values, and Kappa statistics for model accuracy evaluation across three assessment methods.

The accuracy of the evaluated model was categorized into five levels: failure, poor, average, good, and very good. Specifically, intervals for AUC values were defined as follows: (AUC $\leqslant$ 0.60, failure), (0.60 < AUC $\leqslant$ 0.70, poor), (0.70 < AUC $\leqslant$ 0.80, average), (0.80 < AUC $\leqslant$ 0.90, good), and (0.90 < AUC $\leqslant$ 1.00, very good). Similarly, Kappa values were categorized into intervals as follows: (0.40 < Kappa $\leqslant$ 0.55, poor), (0.55 < Kappa $\leqslant$ 0.70, fair), (0.70 < Kappa $\leqslant$ 0.85, good), and (0.85 < Kappa $\leqslant$ 1.00, very good).

In this study, we implemented trial models with different parameter settings to evaluate performance, optimizing these models based on the observed response to known distribution points of *S. italica* and their corresponding environmental factors. The adjustment process involved establishing the range of regularization multipliers (RM) from 0.5 to 4. Additionally, six feature combinations (FC) were employed to optimize the model parameters: L (linear features), LQ (linear features + quadratic features), H (hinge features), LQH (linear features + quadratic features + hinge features), LQHP (linear

features + quadratic features + hinge features + product features), and LQHPT (linear features + quadratic features + hinge features + product features + threshold features). After careful evaluation, the optimal combination for the regularization multiplier and feature selection was determined to be RM and LQHPT, respectively.

2.4 Classification of potentially suitable areas

The MaxEnt model produced an ASCII raster layer depicting the probability (P) of *S. italica* presence, ranging from 0 to 1. To categorize these probabilities into habitat suitability classes, we followed the IPCC-CMIP6 guidelines [21, 14]:

- A probability greater than 0.5 corresponds to a most suitable habitat.
- The range of $0.3 \leqslant P < 0.5$ designates a moderately suitable habitat.
- For probabilities within $0.1 \leqslant P < 0.3$, the habitat is considered low suitable.

3. Results and analysis

3.1 Model accuracy evaluation

Based on the results (Fig. 5), we can conclude that the MaxEnt model predicted an "excellent" AUC value, and the Kappa and the TSS statistics indicated "good" performance; These evaluations confirm the model's appropriate configuration and high reliability for subsequent analysis.

Fig. 5. AUC, Kappa and TSS values of MaxEnt

3.2 Current potential geographical distributions of *S. italica*

The potential suitable habitats for *S. italica* covered an expansive area of 5535.44×10^4 km^2 (Fig. 8), prominently located in the United States (North America), Brazil (South America), Australia (Oceania), China, India (Asia), and the Russian Federation (Europe) (Fig. 6). Within this distribution, high suitable habitats occupied 522.64×10^4 km^2, constituting 9.44% of the total (Fig. 8). Notably, these high suitable habitats were concentrated in the United States, India, and the Russian Federation (Fig. 6). Moderately suitable habitats extended over an area of 1313.75×10^4 km^2, accounting for 23.73% of the total suitable areas (Fig. 8). These habitats were primarily observed in the Russian Federation, United States, China, and India (Fig. 6). Low suitable habitats comprised the largest portion, covering 3699.05×10^4 km^2, representing 66.83% of the total suitable areas (Fig. 8). These areas were predominantly found in China, the United States, the Russian Federation, Brazil, Australia, and other countries (Fig. 6). In summary, the potential geographic range of *S. italica*, as predicted by the MaxEnt model (Fig. 6), surpassed its modern geographic range, illustrating the extensive potential distribution of the species.

Fig. 6. Current global geographical distributions of *S. italica*

3.3 Future potential geographical distributions of *S. italica*

Compared with the current climate conditions, the potentially suitable habitats of *S. italica* drastically reduced under four different emission scenarios (SSP1-2.6, SSP2-4.5, SSP3-7.0 and SSP5-8.5) in the 2050s and 2070s (Fig. 7). Under SSP3-7.0, we identified the maximum reduction at all levels of suitable habitats for both periods, followed by SSP2-4.5, SSP1-2.6, and SSP5-8.5 (Figs. 7, 8).

Fig. 7. Potential global geographical distribution of *S. italica* in the 2050s and 2070s. (a) 2050s,

SSP1-2.6; (b) 2050s, SSP2-4.5g; (c) 2050s, SSP3-7.0; (d)2050s SSP5-8.5; (e) 2070s, SSP1-2.6; (f) 2070s, SSP2-4.5g; (g) 2070s, SSP3-7.0; (h)2070s SSP5-8.5.

Under the SSP3-7.0 scenario, our analysis indicates a substantial reduction in total suitable areas for *S. italica*. By 2050, a predicted decrease of 64.62% is anticipated, further reducing to 64.20% by the 2070s. Highly suitable areas are particularly affected, with an expected decline of 99.51% (2050s) and 99.46% (2070s). Moderately suitable areas will also experience a significant reduction, with a decrease of 84.69% (2050s) and 84.74% (2070s). Conversely, lower suitable areas will undergo a comparatively minimal impact, with a predicted reduction of 52.56% (2050s) and 51.19% (2070s).

Similarly, under the SSP1-2.6 scenario, a noteworthy reduction in suitable habitats is anticipated, with a decrease of 55.86% (2050s) and 51.74% (2070s). The most suitable habitats will witness a more substantial decline, with a decrease of 83.49% (2050s) and 83.51% (2070s). Medium and low-suitable habitats will also be affected, experiencing cuts of 67.29% and 41.00% in the 2050s and 66.67% and 41.95% in the 2070s, respectively.

Under SSP2-4.5, a decrease in total suitable habitats by 44.25% (2050s) and 43.60% (2070s) is expected. Highly suitable habitats will be most affected, with a reduction of 77.56% (2050s) and 76.74% (2070s). Medium and low suitable zones will also decrease by 61.45% and 33.44% in the 2050s and 61.13% and 32.69% in the 2070s, respectively.

Lastly, with the SSP5-8.5 scenario, a predicted reduction of 44.25% in total suitable habitats is anticipated in the 2050s and 43.60% in the 2070s. Highly suitable areas will again be most affected, with a decrease of 77.56% (2050s) and 76.74% (2070s). Medium suitable habitats will see a significant decline of 61.45% in the 2050s and 61.13% in the 2070s, while lower suitable habitats should be reduced by 33.44% (2050s) and 32.69% (2070s).

In summary, climate change is expected to significantly impact the suitable habitats of *S. italica*, leading to a loss of potential habitats in the coming decades under various emission scenarios. This transformation is characterized by the conversion of highly suitable areas into medium and low-suitable areas, as well as the shift of medium-suitable habitats towards low-suitable and unsuitable habitats (Figs. 7 and 8).

Fig. 8. Suitable areas for *S. italica* under different climate change scenarios(10^4 km^2)

3.4 Combinations of dominant environmental variables affecting the distribution of *S. italica*

The determination of dominant factors in environmental studies is marked by varying perspectives among researchers. Presently, a prevalent approach involves establishing dominance based on the contribution rate of environmental factors. This entails considering factors as dominant once they surpass a certain contribution rate threshold. However, the diversity in criteria arises due to the subjective nature of threshold selection.

In this study, we adopted a specific criterion for identifying dominant environmental factors. The top three environmental factors with the highest contribution rates were selected as dominant factors, as illustrated in Figure 9. Notably, across different periods and under various emission background concentrations, the contribution rates of soil factor, driest month precipitation (Bio14), and hottest season precipitation (Bio18) consistently ranked much higher than other variables, securing the top

three positions. Consequently, these three factors were collectively utilized as the dominant data in this study, offering a focused and robust basis for the analysis.

Fig. 9. Environmental variables and their contributions of *S. italica* (a) 2050s, SSP1-2.6; (b) 2050s, SSP2-4.5g; (c) 2050s, SSP3-7.0; (d)2050s SSP5-8.5; (e) 2070s, SSP1-2.6; (f) 2070s, SSP2-4.5g; (g) 2070s, SSP3-7.0; (h)2070s SSP5-8.5; (i) current.

The assessment of relationships between the presence probability of *S. italica* and environmental factors was conducted through the analysis of response curves for each environmental variable. In this analysis, when the presence probability surpassed 0.5, it signified that the corresponding environmental factor value favored the growth of *S. italica*. To provide a more intuitive depiction of the influence of environmental variables on the distribution of *S. italica*, the response curve for the combination of dominant environmental variables in this study was derived, as illustrated in Figure 10. This graphical representation offers insights into how the probability of *S. italica* presence responds to variations in the combined values of the selected dominant environmental factors.

Fig. 10. Response curves of existence probability of *S. italica*

4. Discussion

4.1 Changes in the potential geographic distribution of *S. italica* under future climate change scenarios

In this study, the MaxEnt model was employed to predict changes in the potential global geographic distribution of *S. italica*, utilizing environmental variables from both current and future climate scenarios under four different emission scenarios (Fig. 10). The model projections indicate that the potential geographic distribution of *S. italica* in the 2050s and 2070s under the four emission scenarios is expected to be lower compared to the potential distribution under modern climate conditions.

The suitability of areas for *S. italica* exhibited a trend of both increasing and decreasing with rising precipitation. This trend may be attributed to precipitation levels exceeding the threshold suitable for the survival of *S. italica* in many regions, leading to a decrease in its probability of survival, especially in the SSP3-7.0 emission scenario where temperature and precipitation values were outside the suitable ranges. This aligns with findings from Thomas et al., who studied the extinction risk of organisms on a sample area covering 20% of the Earth's surface, suggesting varying impacts on different species in response to climate change [26]. While some species benefit from climate warming, the impact on *S. italica* is predominantly negative.

The suitable habitats of *S. italica* in the 2050s and 2070s show a contraction to the west in Europe, a trend to the north in Africa, and a trend towards the south in Asia, North America, South America, and Oceania. The SSP5-8.5 emission scenario in the 2070s indicates the smallest loss of suitable habitats for *S. italica*, covering an area of 2369.53 × 104 km2 (Fig. 10). Conversely, the largest loss of suitable habitat occurs in the 2050s under the SSP3-7.0 emission scenario, with an area of 3576.80 × 104 km2 (Fig. 10). China, the United States, Brazil, and Australia are highlighted as regions experiencing the most significant loss.

Under the SSP5-8.5 emission scenario, *S. italica* is projected to experience the least reduction in suitable habitats at all levels. This resilience is attributed to the species' ability to tolerate high temperatures and drought, making it less vulnerable to this particular emission scenario. These results

align with other studies examining the response of various species to changing climate conditions.

It's important to note that climate change could indirectly affect the population and distribution characteristics of *S. italica* by influencing the ecosystem directly. Additionally, irrational human activities, such as urban construction, hydroelectric development, and other industrial practices, could further contribute to a dramatic decline in potential distribution areas. The study's focus on two time periods, 2050 and 2070, for environmental factor variables suggests the potential for future research to consider multiple periods to derive an overall trend in the species' potential geographic distribution in response to climate changes.

Fig. 11. Changes in the potential geographical distribution of *S. italica* under climate change scenarios in the future

4.2 Constraints of environmental variables on the potential geographical distribution of *S. italica*

The MaxEnt model results highlight soil and precipitation factors, specifically the driest monthly precipitation and hottest seasonal precipitation, as critical environmental factors limiting the potential geographic distribution of *S. italica*. The study reveals that the probability of *S. italica* presence is influenced by variations in these precipitation factors, with increased driest-month precipitation and hottest-season precipitation positively impacting the probability of existence. This aligns with previous research by Zhang and Yang, demonstrating the sensitivity of *S. italica* growth to precipitation levels, both low and excessive [40, 34]. The study corroborates findings by Zhang, emphasizing the substantial impact of moisture conditions on *S. italica* yield, projecting a significant influence on the species' probability of existence with future precipitation increments [39].

Cao's study, based on actual weather station data, supports the conclusion that climatic variables such as driest monthly precipitation and hottest seasonal precipitation play a primary role in constraining the growth and distribution of *S. italica* [22]. These results are consistent with the findings of this study. Further, the influence of soil factors on *S. italica* growth and development is supported by research conducted by Kaur, Bandyopadhyay, and Nissi, highlighting the significance of soil fertility and microorganisms in soil as important environmental factors for *S. italica* [13, 3, 23].

Historical studies by Dong and Chen, exploring the development and decline of *S. italica* cultivation over millennia, underscore the importance of temperature as a significant constraint on the potential geographical distribution of *S. italica* [9, 8]. These findings align with the temperature-related factors identified in the MaxEnt model predictions.

The study focuses on predicting the potential geographic distribution of *S. italica* in China and identifies the environmental variables limiting its potential distribution. Changes in the study area over time may impact the range of environmental factors influencing *S. italica* growth. While other environmental factors, such as vegetation cover, could influence the species' potential distribution, they were not included in the study due to challenges in accurately predicting global vegetation cover in the future. Therefore, the results obtained should be applied in practice with consideration for local hydrogeological conditions. Overall, the study contributes valuable insights to the global macro plan for the rational cultivation of *S. italica*.

4.3 The importance of conducting simulations of the potential geographic distribution of *S. italica*

Global climate change poses a serious threat to agricultural production, directly impacting food security and, consequently, human development. The food crisis, stemming from climate change, has a far-reaching impact, making it one of the most critical challenges globally [2, 37]. The ecological niche models used in this study to project the potential geographic distribution of *S. italica* contribute to a clearer understanding of how this crop responds to climate change. As a significant global miscellaneous crop, *S. italica* plays a crucial role in ensuring food security and human health.

The study's predicted results indicate that the potential geographic distribution of *S. italica* is adversely affected in the context of climate change. These findings serve as a crucial first step in the macro plan for the rational cultivation of *S. italica*. To address these challenges, promoting the planting of *S. italica* in highly suitable areas, especially in the context of grassland restoration, is recommended. Increasing knowledge and implementing practices for controlling pests and diseases affecting *S. italica* are essential. Immediate actions, such as quarantine measures for affected crops and treatment of diseases and insect infestations, are crucial. Through the application of scientific knowledge and effective control measures, it is possible to mitigate the impact of climate change on *S. italica* cultivation, contributing to broader food security efforts.

5. Conclusions

The simulation of the potential geographical distribution of *S. italica* based on current climate conditions indicates its main distribution areas include the United States (North America), Brazil (South America), Australia (Oceania), China, India (Asia), and the Russian Federation (Europe). However, under future climate change scenarios, there is a significant reduction in the potential distribution areas of *S. italica*. This reduction could have unfavorable implications for the future cultivation of *S. italica*. The critical environmental factors identified as limiting the potential geographical distribution of *S. italica* include soil and precipitation factors, specifically the driest monthly precipitation and hottest seasonal precipitation. In conclusion, the study suggests that climate change has the potential to significantly impact the future distribution pattern of *S. italica* cultivation, leading to a reshaping of production and trade patterns. This insight is valuable for anticipating and addressing challenges in *S. italica* cultivation in the face of changing climate conditions.

Funding

The Scientific research initiation project of Mianyang Normal University (QD2019A13, QD2021A37 and QD2023A01), the Funding of the Open Project from the Ecological Security and Protection Key Laboratory of Sichuan Province (ESP1608, ESP2008, ESP2201 and ESP2204), the Sichuan Provincial Education Department Scientific Research Project (15ZB0283), the Sichuan Provincial Science and Technology Department Project (2023NSFSC0750).

Acknowledgments

We sincerely thank Zhangqiang You in the Ecological Security and Protection Key Laboratory of Sichuan Province for providing us with financial and technical support in this study

References

[1] Alonso N, Perez-Jorda G. The Origins of Millet Cultivation (Panicum miliaceum and Setaria italica) along Iberia's Mediterranean Area from the 13th to the 2nd Century BC. Agronomy-Basel 2023;13(2):584.

[2] Asseng S, Ewert F, Martre P, Rotter RP, Lobell DB, Cammarano D, et al. Rising temperatures reduce global wheat production. Nature Climate Change 2015;5(2):143-7.

[3] Bandyopadhyay T, Swarbreck SM, Jaiswal V, Maurya J, Gupta R, Bentley AR, et al. GWAS identifies genetic loci underlying nitrogen responsiveness in the climate resilient C4 model Setaria italica (L.). Journal of Advanced Research 2022;42:249-61.

[4] Behera D, Swain A, Karmakar S, Dash M, Swain P, Baig MJ, et al. Overexpression of Setaria italica phosphoenolpyruvate carboxylase gene in rice positively impacts photosynthesis and agronomic traits. Plant Physiology and Biochemistry 2023;194:169-81.

[5] Bo W, Fangfang S, Xin M, HUANG Tingting, Songmei M. Possible response of the suitable distribution areas of endangered desert Plant Gymnocarpos przewalskii to future climate change scenario. Journal of Shihezi University(Natural Science) 2019;37(04):490-7.

[6] Brooks TM, Mittermeier RA, Da FG, Gerlach J, Hoffmann M, Lamoreux JF, et al. Global biodiversity conservation priorities. Science 2006;313(5783):58-61.

[7] Cao B, Bai C, Zhang M, Lu Y, Gao P, Yang J, et al. Future landscape of renewable fuel resources: Current and future conservation and utilization of main biofuel crops in China. Science of the Total Environment 2022;806.

[8] Chen FH, Dong GH, Zhang DJ, Liu XY, Jia X, An CB, et al. Agriculture facilitated permanent human occupation of the Tibetan Plateau after 3600 BP. Science 2015;347(6219):248-50.

[9] Dong G, Li R, Lu M, Zhang D, James N. Evolution of human-environmental interactions in China from the Late Paleolithic to the Bronze Age. Progress in Physical Geography-Earth and Environment 2020;44(2):233-50.

[10] Elith J, Graham CH, Anderson RP, Dudik M, Ferrier S, Guisan A, et al. Novel methods improve prediction of species' distributions from occurrence data. Ecography 2006;29(2):129-51.

[11] Huang Y, Zeng Y, Jiang P, Chen H, Yang J. Prediction of Potential Geographic Distribution of Endangered Relict Tree Species Dipteronia sinensis in China Based on MaxEnt and GIS. Polish Journal of Environmental Studies 2021;31(4):3597-609.

[12] Janeth Martinez-Macias K, Yuridia Marquez-Guerrero S, Rafael Martinez-Sifuentes A, Angel Segura-Castruita M. Habitat Suitability of Fig (Ficus carica L.) in Mexico under Current and Future Climates. Agriculture-Basel 2022;12(11).

[13] Kaur T, Devi R, Kumar S, Kour D, Yadav AN. Synergistic Effect of Endophytic and Rhizospheric Microbes for Plant Growth Promotion of Foxtail Millet (Setaria italica L.). National Academy Science Letters-India 2023;46(1):27-30.

[14] Lan R, Chen J, Pan J, Chen R, Lin H, Li Z, et al. Simulation of Potential Suitable Distribution of Endangered Medicinal of Paeonia rockii under Climate Change Scenarios. Polish Journal of Environmental Studies 2023;32(3):2181-97.

[15] Lan R, Chen R, Lin H, Huang Y, Wang R. Suitable Area of Invasive Species Alexandrium under Climate Change Scenarios in China Sea Areas. Polish Journal of Environmental Studies 2023;32(2):1199-217.

[16] Larson G, Piperno DR, Allaby RG, Purugganan MD, Andersson L, Arroyo-Kalin M, et al. Current perspectives and the future of domestication studies. Proceedings of the National Academy of Sciences of the United States of America 2014;111(17):6139-46.

[17] Leng W, He HS, Bu R, Dai L, Hu Y, Wang X. Predicting the distributions of suitable habitat for three larch species under climate warming in Northeastern China. Forest Ecology and Management 2008;254(3):420-8.
[18] Li J, Fan G, He Y. Predicting the current and future distribution of three Coptis herbs in China under climate change conditions, using the MaxEnt model and chemical analysis. Science of the Total Environment 2020;698.
[19] Li W, Xu Z, Shi M, Chen J. Prediction of potential geographical distribution patterns of Salix tetrasperma Roxb. in Asia under different climate scenarios. Acta Ecologica Sinica 2019;39(9):3224-34.
[20] Liu L, Guan L, Zhao H, Huang Y, Mou Q, Liu K, et al. Modeling habitat suitability of Houttuynia cordata Thunb (Ceercao) using MaxEnt under climate change in China. Ecological Informatics 2021;63.
[21] Liu L, Zhang Y, Huang Y, Zhang J, Mou Q, Qiu J, et al. Simulation of potential suitable distribution of original species of Fritillariae Cirrhosae Bulbus in China under climate change scenarios. Environmental Science and Pollution Research 2022;29(15):22237-50.
[22] Mingyu C. Gis based ecological suitability millet plantingzoning research in Heilongjiang province. Heilongjiang Bayi Agricultural University; 2022.
[23] Nissi WI, Debbarma V. Effect of Organic Manures and Fish Amino Acid on Growth and Yield of Foxtail Millet (Setaria italica L.). International Journal of Plant & Soil Science 2022;34(23):601-7.
[24] Santana PA, Jr., Kumar L, Da Silva RS, Pereira JL, Picanco MC. Assessing the impact of climate change on the worldwide distribution of Dalbulus maidis (DeLong) using MaxEnt. Pest Management Science 2019;75(10):2706-15.
[25] Song H, Wang T, Li L, Xing L, Xie HF, Feng BL, et al. Comparative transcriptome analysis provides insights into grain filling commonalities and differences between foxtail millet Setaria italica (L.) P. Beauv. varieties with different panicle types. PeerJ 2022;10.
[26] Thomas CD, Cameron A, Green RE, Bakkenes M, Beaumont LJ, Collingham YC, et al. Extinction risk from climate change. Nature 2004;427(6970):145-8.
[27] Thuiller W, Lavorel S, Araújo MB, Sykes MT, Prentice IC. Climate change threats to plant diversity in Europe. Proceedings of the National Academy of Sciences 2005;102(23):8245-50.
[28] Victoria Lantschner M, de la Vega G, Corley JC. Predicting the distribution of harmful species and their natural enemies in agricultural, livestock and forestry systems: an overview. International Journal of Pest Management 2019;65(3):190-206.
[29] Xian X, Zhao H, Wang R, Huang H, Chen B, Zhang G, et al. Climate change has increased the global threats posed by three ragweeds (Ambrosia L.) in the Anthropocene. Science of the Total Environment 2023;859(Pt 2):160252-.
[30] Xu W, Miao Y, Zhu S, Cheng J, Jin J. Modelling the Geographical Distribution Pattern of Apple Trees on the Loess Plateau, China. Agriculture-Basel 2023;13(2).
[31] Xu W, Zhu S, Yang T, Cheng J, Jin J. Maximum Entropy Niche-Based Modeling for Predicting the Potential Suitable Habitats of a Traditional Medicinal Plant (Rheum nanum) in Asia under Climate Change Conditions. Agriculture-Basel 2022;12(5).
[32] Yang J-T, Jiang X, Chen H, Jiang P, Liu M, Huang Y. Predicting the Potential Distribution of the Endangered Plant Magnolia wilsonii Using MaxEnt under Climate Change in China. Polish Journal of Environmental Studies 2022;31(5):4435-45.

[33] Yang J, Huang Y, Jiang X, Chen H, Liu M, Wang R. Potential geographical distribution of the edangred plant Isoetes under human activities using MaxEnt and GARP. Global Ecology and Conservation 2022;38.

[34] Yang X, Tian Q, Yan J, Chen Y. Characterizing Root Morphological Traits in 65 Genotypes of Foxtail Millet (Setaria italica L.) from Four Different Ecological Regions in China. Agronomy-Basel 2022;12(6).

[35] Yu X, Tao X, Liao J, Liu S, Xu L, Yuan S, et al. Predicting potential cultivation region and paddy area for ratoon rice production in China using Maxent model. Field Crops Research 2022;275.

[36] Yuan M, Zheng N, Yang Y, Liu C. Robust optimization for sustainable agricultural management of the water-land-food nexus under uncertainty. Journal of Cleaner Production 2023;403.

[37] Yue Y, Zhang P, Shang Y. The potential global distribution and dynamics of wheat under multiple climate change scenarios. Science of the Total Environment 2019;688:1308-18.

[38] Zhang H, Zhao H, Wang H. Potential geographical distribution of populus euphratica in China under future climate change scenarios based on Maxent model. Acta Ecologica Sinica 2020;40(18):6552-63.

[39] Zhang W, Wang B, Liu B, Chen Z, Lu G, Bai C, et al. The Effects of Meteorological Factors on Grain Yield of Foxtail Millet (Setaria italica Beauv.) under Different Water Supply Conditions. Crops 2023;3(1):53-62.

[40] Zhang W, Wang B, Liu B, Chen Z, Lu G, Ge Y, et al. Trait Selection for Yield Improvement in Foxtail Millet (Setaria italica Beauv.) under Climate Change in the North China Plain. Agronomy-Basel 2022;12(7).

[41] Zhao H, Xian X, Yang N, Zhang Y, Liu H, Wan F, et al. Insights from the biogeographic approach for biocontrol of invasive alien pests: Estimating the ecological niche overlap of three egg parasitoids against Spodoptera frugiperda in China. Science of the Total Environment 2023;862.

[42] Zhao Y, Li Y, Zhen X, Zhang J, Zhang Q, Liu Z, et al. Uncovering the mechanism of anthocyanin accumulation in a purple-leaved variety of foxtail millet (Setaria italica) by transcriptome analysis. PeerJ 2022;10.

[43] Zhao Z. New Archaeobotanic Data for the Study of the Origins of Agriculture in China. Current Anthropology 2011;52:S295-S306.

[44] Zheng N. Comparative analysis of nutrition function components and antioxidation effect in millets and proso millets. Tianjin University of Science & Technology; 2018.

Figure1 Click here to access/download;Figure;Fig 1.png

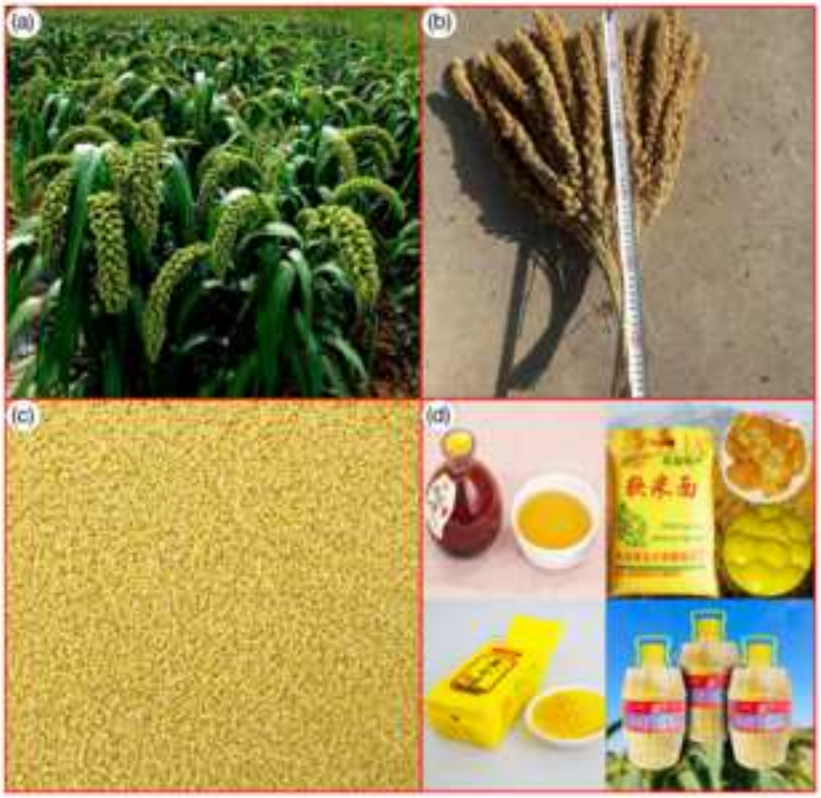

 Click here to access/download;Figure;Fig 2.png

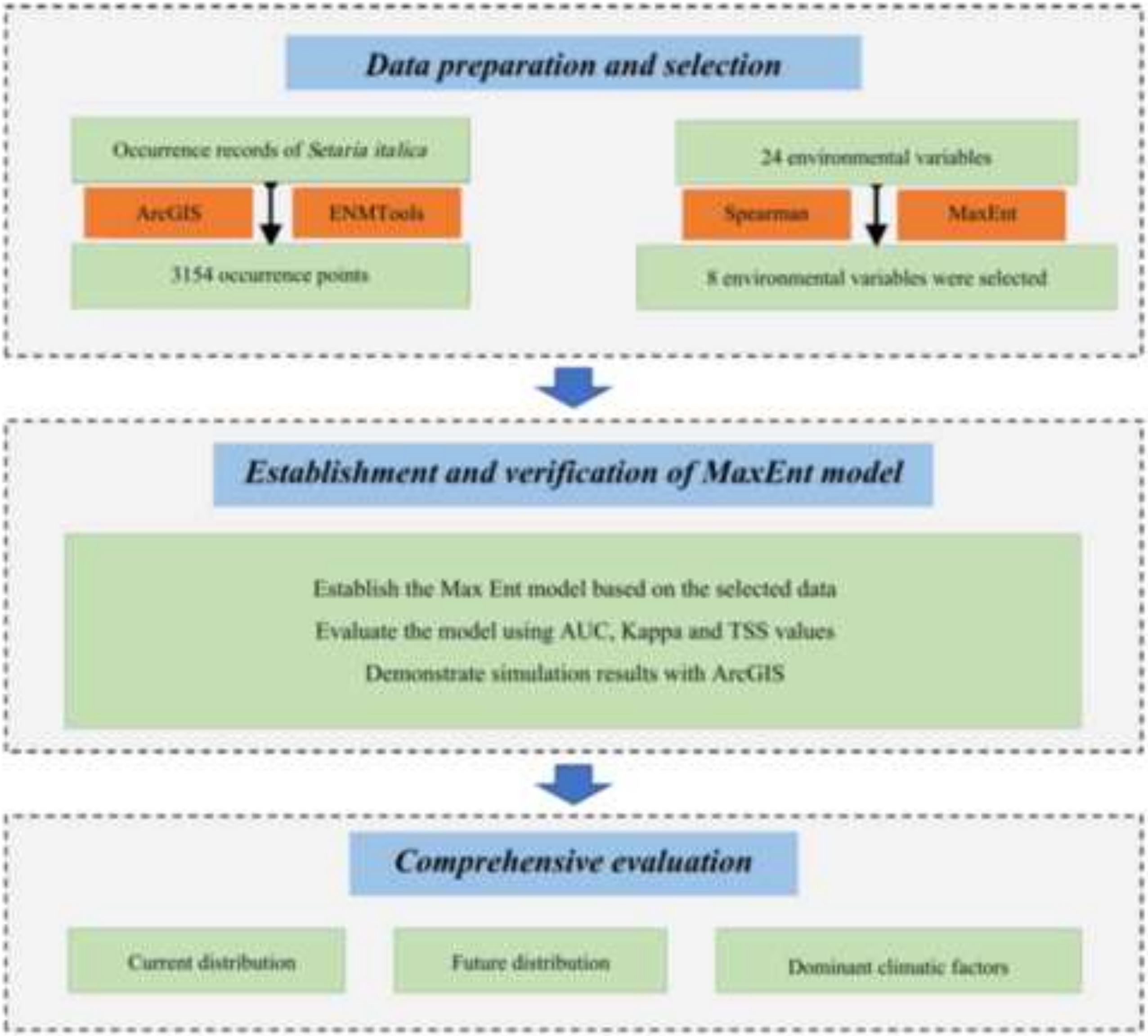

Figure3

Click here to access/download;Figure;Fig 3.png

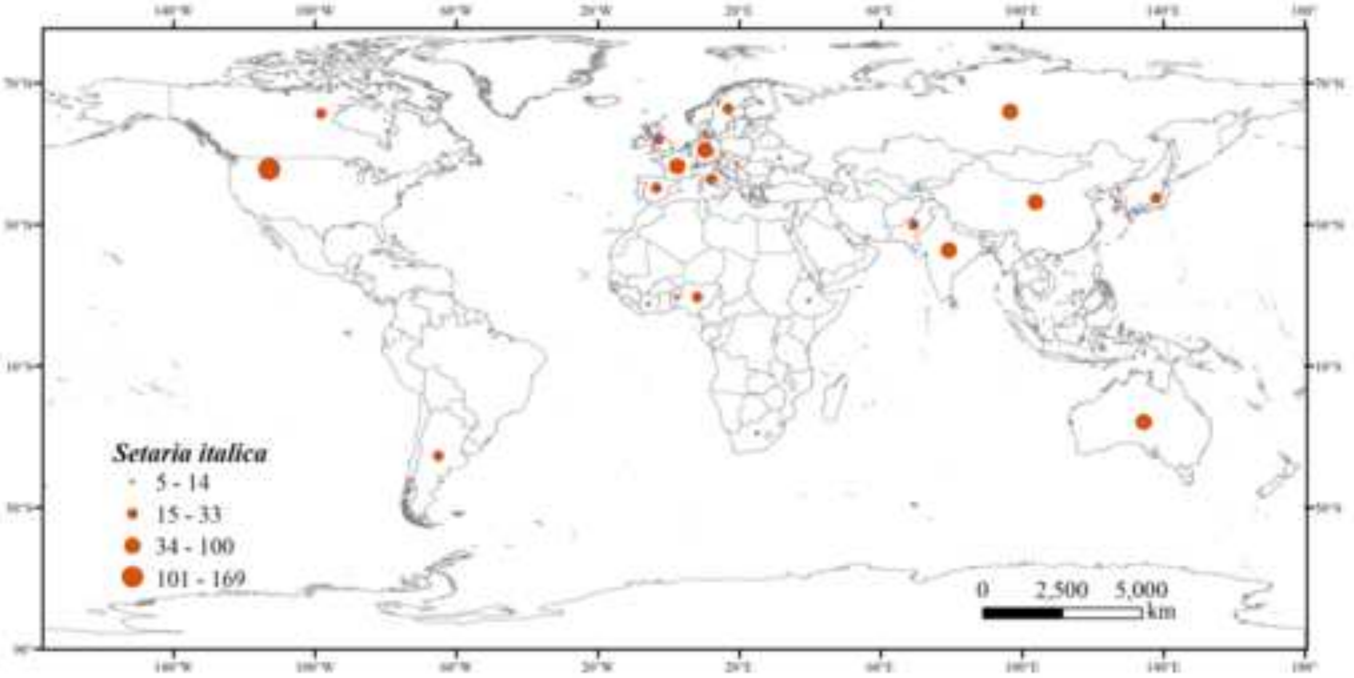

Figure4

Click here to access/download;Figure;Fig 4.png

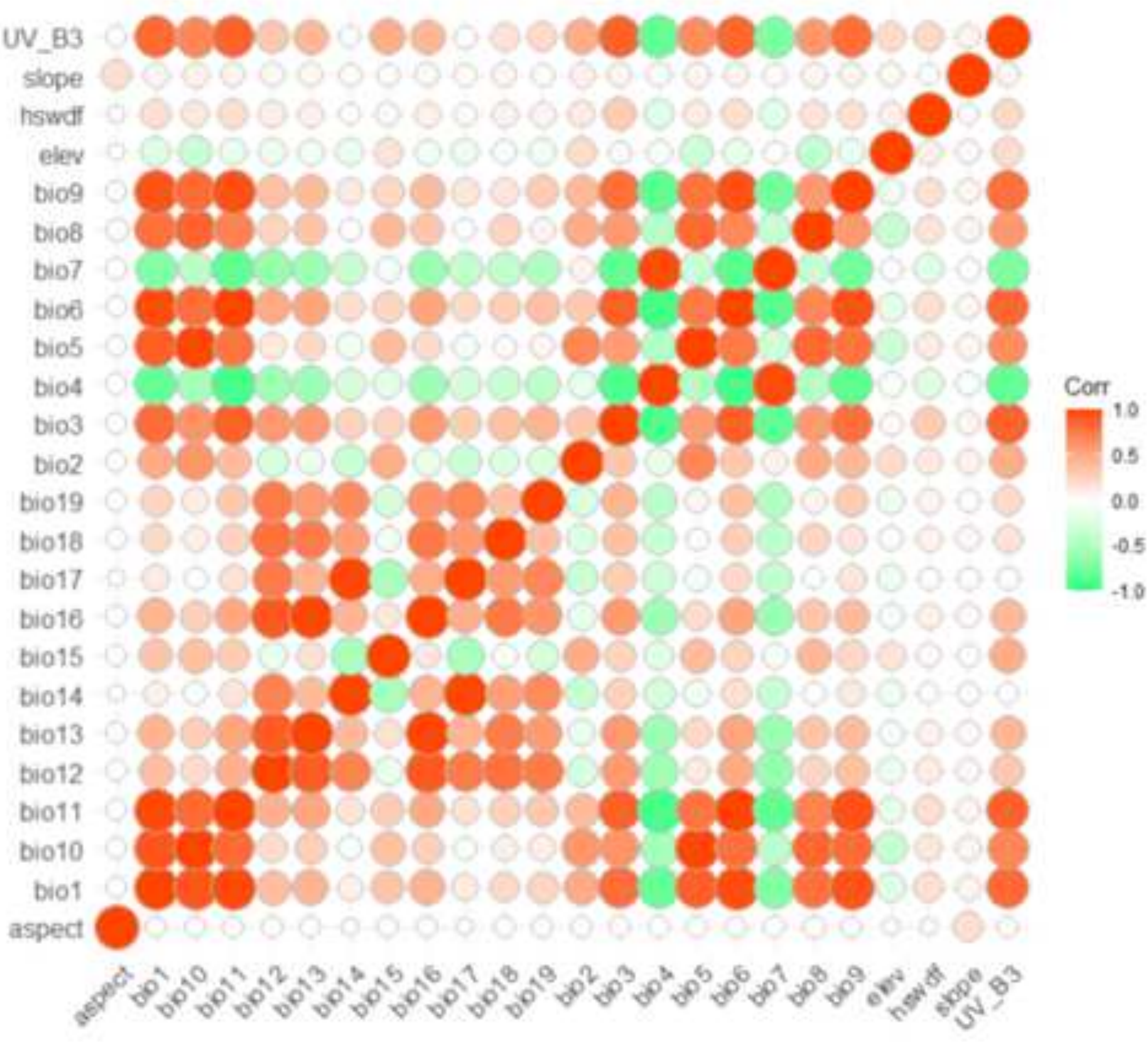

Figure5

Click here to access/download;Figure;Fig 5.jpg

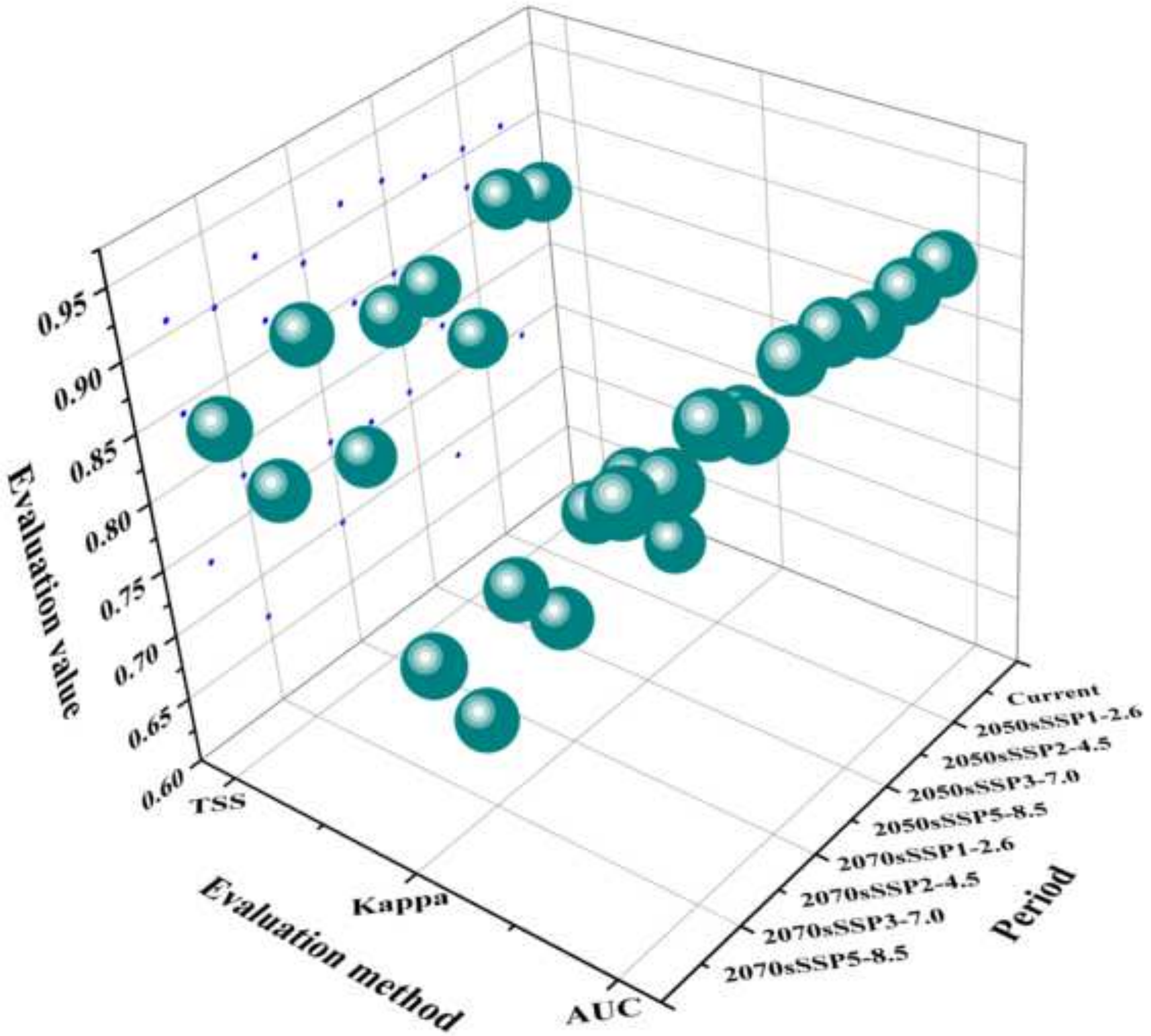

Figure6

Click here to access/download;Figure;Fig 6.png

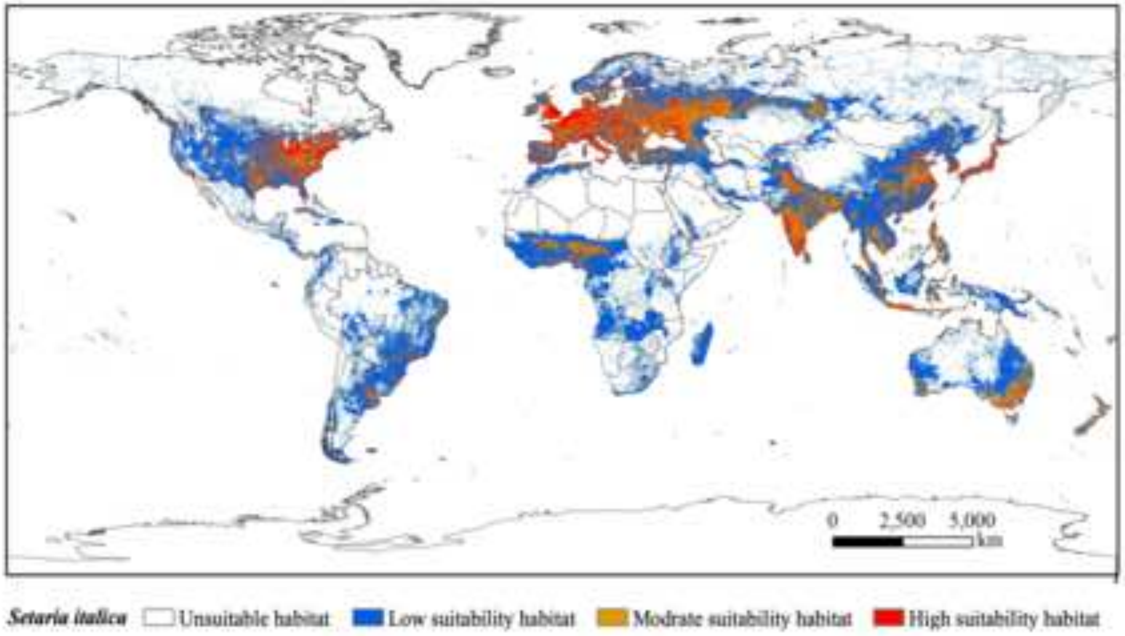

 Click here to access/download;Figure;Fig 7.png

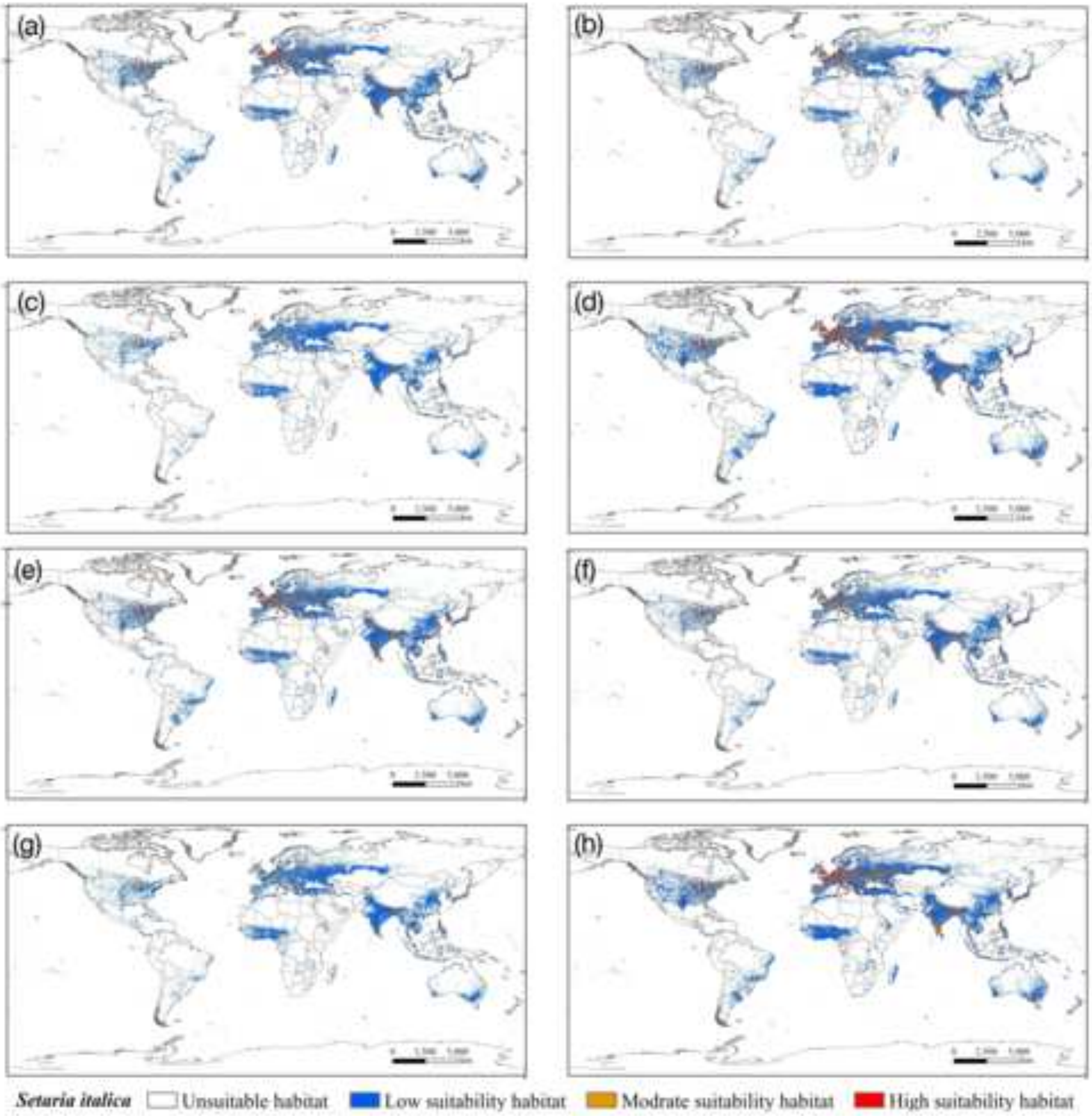

Figure8

Click here to access/download;Figure;Fig 8.png

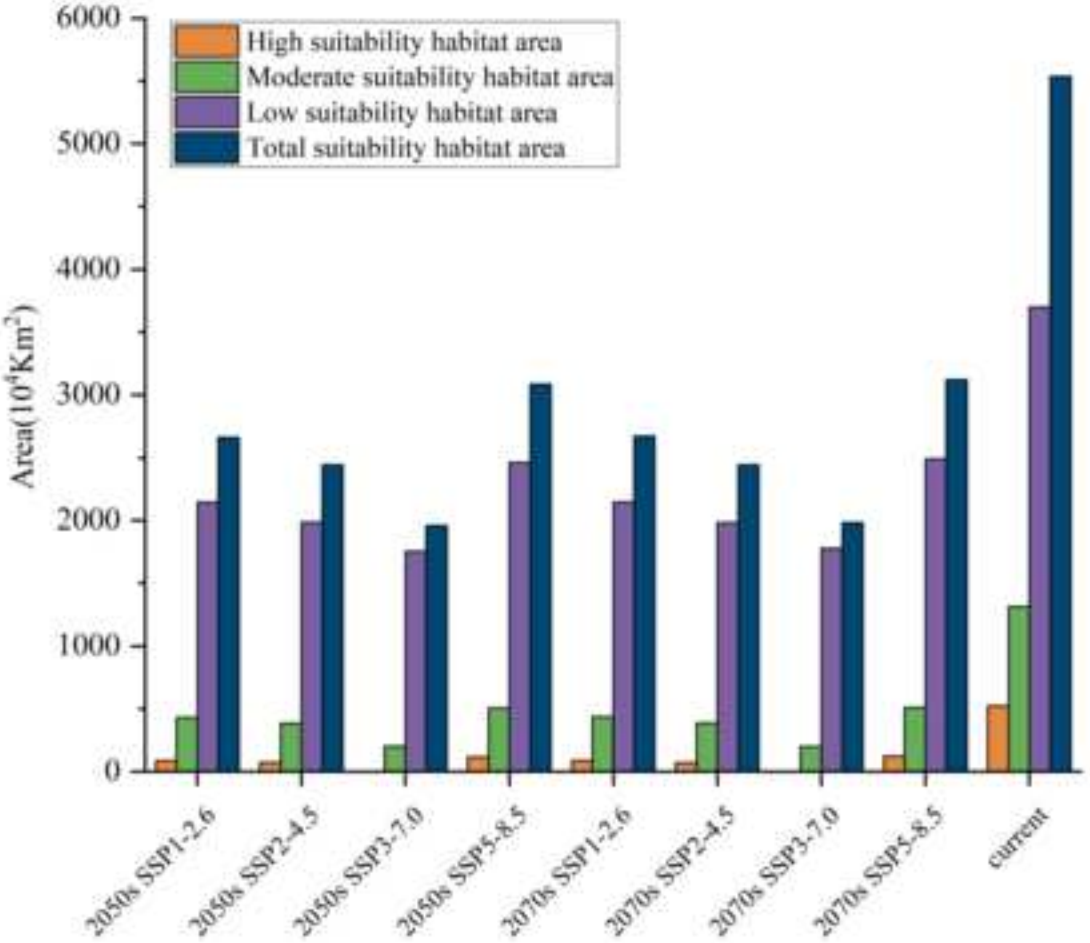

Figure9

Click here to access/download;Figure;Fig 9.png

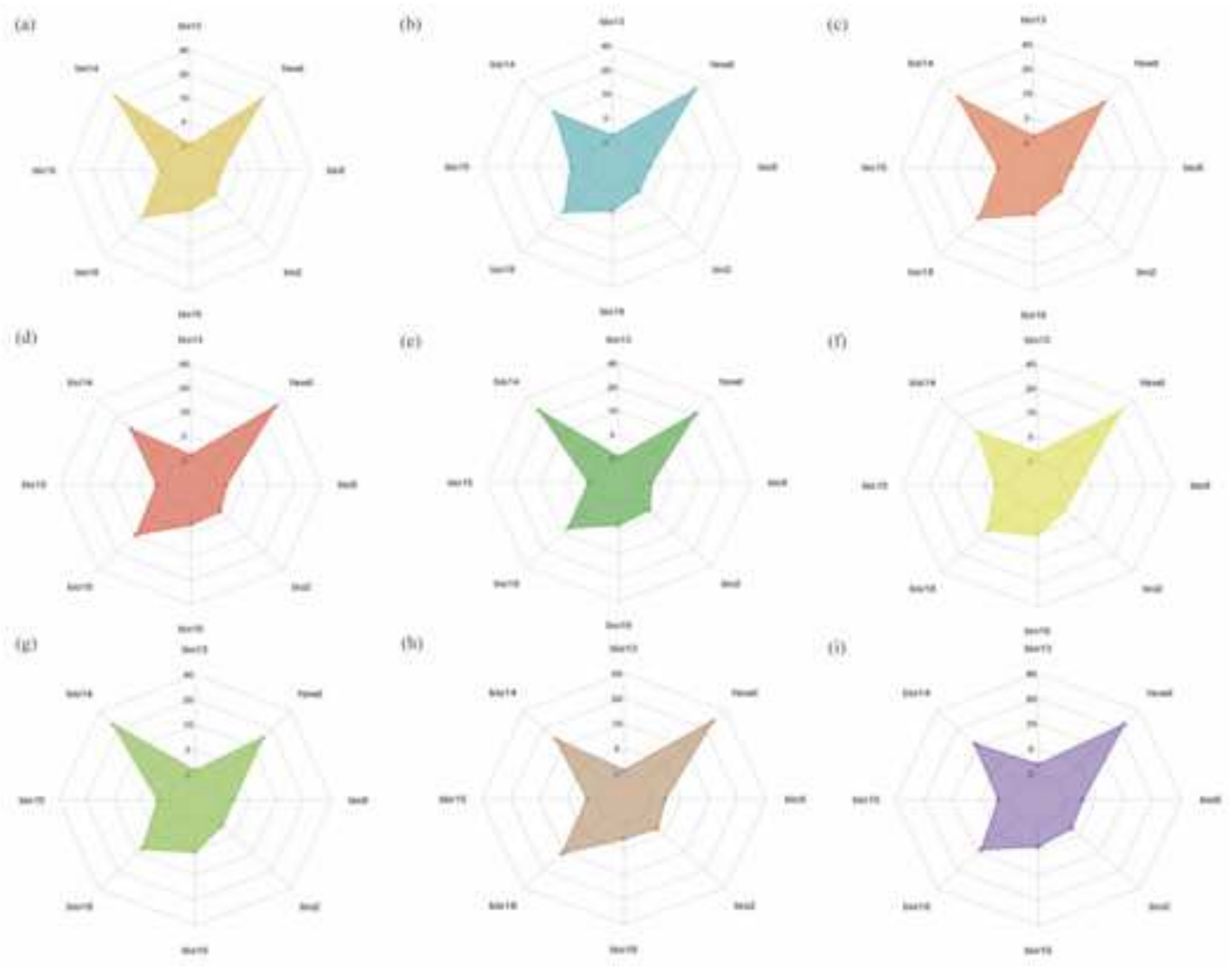

Figure10 Click here to access/download;Figure;Fig 10.png

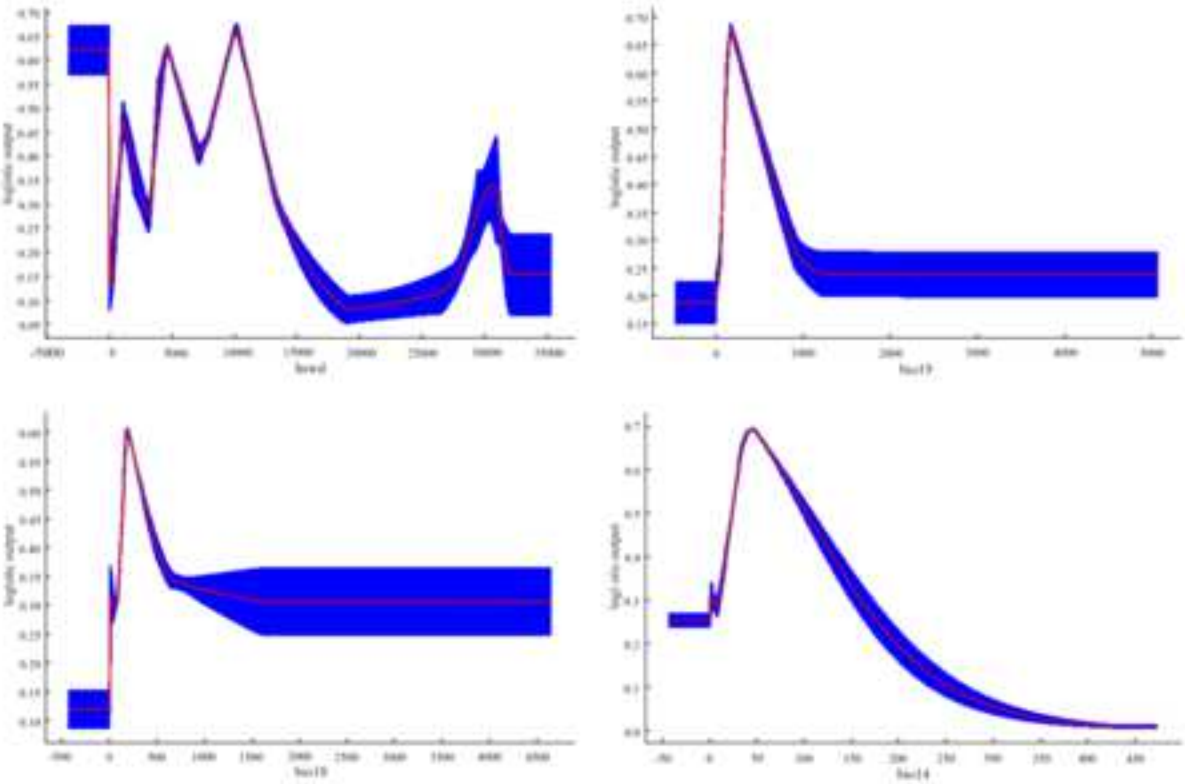

Figure11 Click here to access/download;Figure;Fig 11.png

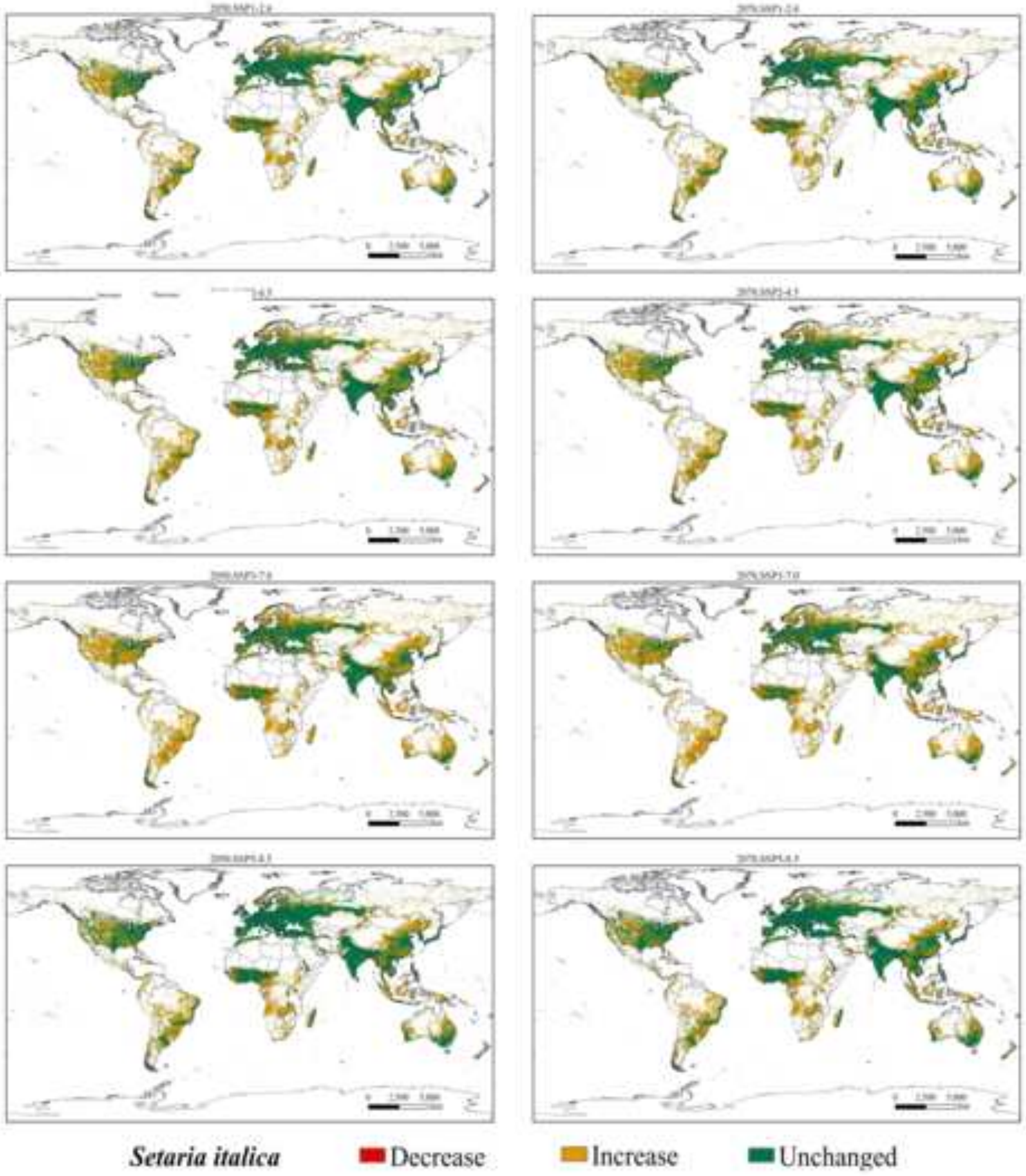

Supplement: Attachment — Submitted filename: PONE-D-23-42450_reviewer.pdf [file pone.0301751.s003.pdf]
